# The Plant Ontology^™^ Consortium and Plant Ontologies

**DOI:** 10.1002/cfg.154

**Published:** 2002-04

**Authors:** The Plant Ontology Consortium

**Affiliations:** Department of Agronomy University of Missouri-Columbia Columbia Missouri 65211-7020 USA

## Abstract

The goal of the Plant Ontology^™^ Consortium is to produce structured controlled vocabularies, arranged in ontologies, that can be applied to plant-based database information even as knowledge of the biology of the relevant plant taxa (e.g. development, anatomy, morphology, genomics, proteomics) is accumulating and changing. The collaborators of the Plant Ontology^™^ Consortium (POC) represent a number of core participant database groups. The Plant Ontology^™^ Consortium is expanding the paradigm of the Gene Ontology^™^ Consortium (http://www.geneontology.org). Various trait ontologies (agronomic traits, mutant phenotypes, phenotypes, traits, and QTL) and plant ontologies (plant development, anatomy [incl. morphology]) for several taxa (*Arabidopsis*, maize/corn/*Zea mays* and rice/*Oryza*) are under development. The products of the Plant Ontology^™^ Consortium will be open-source.


**Comparative and Functional Genomics**
*Comp Funct Genom* 2002; **3**: 137–142.
Published online 15 March 2002 in Wiley InterScience (www.interscience.wiley.com). **DOI:** 10.1002/cfg.154

**Conference Review**

### The Plant Ontology™ Consortium and Plant Ontologies

The Plant Ontology™ Consortium†
*Department of Agronomy, University of Missouri-Columbia, Columbia, Missouri, 65211-7020, USA*
*E-mail: Leszek@missouri.edu*

*†A full list of authors/affiliations/web addresses appears at the end of this paper.*

### Abstract

**The goal of the Plant Ontology™ Consortium is to produce structured controlled vocabularies, arranged in ontologies, that can be applied to plant-based database information even as knowledge of the biology of the relevant plant taxa (e.g. development, anatomy, morphology, genomics, proteomics) is accumulating and changing. The collaborators of the Plant Ontology™ Consortium (POC) represent a number of core participant database groups. The Plant Ontology™ Consortium is expanding the paradigm of the Gene Ontology™ Consortium (http://www.geneontology.org). Various trait ontologies (agronomic traits, mutant phenotypes, phenotypes, traits, and QTL) and plant ontologies (plant development, anatomy [incl. morphology]) for several taxa (*Arabidopsis*, maize/corn/*Zea mays* and rice/*Oryza*) are under development. The products of the Plant Ontology™ Consortium will be open-source.****Copyright © 2002 John Wiley & Sons, Ltd.**

Received: 11 February 2002
Accepted: 12 February 2002

**Keywords:** ***Arabidopsis*; controlled vocabulary; corn; database; maize; ontology; rice**

Plant databases are expanding in number, size and complexity. This is especially true of economically important plant taxa such as maize/corn (*Zea mays*), rice (*Oryza sativa*) and soybean (*Glycine max*) but is also true of taxa regarded as 'model organisms' for plant science research purposes, such as *Arabidopsis thaliana* and rice (*Oryza*). These information-rich databases face the challenge of accurately and consistently documenting features such as gene structures, products and functions, phenotypes, traits, developmental stages and anatomical parts besides other information. It will be increasingly desirable for inter-database queries to be performed between these plant-based databases to exploit comparative genomic strategies to elucidate functional aspects of plant biology and conduct studies of synteny. These databases will facilitate interpolation and extrapolation of data that will facilitate the development of further hypotheses to be tested. However, terms used to describe comparable objects within and between databases are sometimes quite variable and limit the ability to accurately and successfully query information in and across different databases. One solution to this problem involves the development and application of structured controlled vocabularies arranged in ontologies.

### What is an ontology?

An ontology is a classification methodology for formalizing a subject's knowledge in a structured way (typically for consumption by an electronic database). Dictionaries and encyclopedias are examples of ontologies, as are many web-based entities, such as Yahoo and Excite, and so is the schema for a database. A more formal definition of ontology is available from: **http://www-ksl.stanford.edu/onto-std/mailarchive/0136.html**. In the world of structured information, ontologies, comprising structured controlled vocabularies, play a very important role in facilitating information retrieval. This is because the relationships between the controlled vocabulary terms, in the ontologies, must be defined in order to query the information. If the relationships between the terms (objects) are not well structured (biologically accurate) the information retrieved will

Copyright © 2002 John Wiley & Sons, Ltd.

be less valuable. Furthermore, the definitions that accompany the controlled vocabulary terms are very important in that they facilitate the consistent use of the controlled vocabulary terms in database curation.

### Biology-based ontologies

In biology-based ontologies the controlled vocabulary terms are arranged in such a way that their placement reflects the known or putative biological associations between the objects represented by the controlled vocabulary terms. Consequently, considerable effort must be invested into the compilation of the controlled vocabulary terms and the definitions of these terms. The terms and their definitions need to be biologically correct and internationally acceptable. The arrangement of the controlled vocabulary terms in the ontology must reflect the current understanding of the biological relationships between the associated plant parts at the subcellular organelle level, cell level, tissue level and organ level. If the ontology of the relationships between the terms is correct, the information retrieved via a database search is likely to be valuable and correct. The converse is also likely to be true. While it is ideal to use terms that are internationally acceptable this does not imply that local terms and/or synonyms cannot be used. A parsing facility, utilizing synonyms, can be used to overcome this difficulty.

### Ontology structure

It is relatively easy to design an ontology based on concrete facts such as names, birth dates etc. However, it is considerably more difficult to design an ontology based on knowledge that is incomplete or not yet well understood, or that as yet does not have unanimous support. However, the Plant Ontology™ Consortium is attempting to develop various plant ontologies that will represent our current and future understanding of relationships among various plant-based knowledge domains e.g. anatomy and morphology, development, traits. These ontologies will provide open-source, common vocabularies of defined terms. The relationships between concepts (represented by controlled vocabulary terms) within and between ontologies is represented by the use of Directed Acyclic Graphs (DAGs). A DAG is similar to a hierarchical structure but is superior because terms (representing concepts) within a DAG structure have the ability to have one or more than one 'parent'. DAGs are able to represent biological relationships more readily than typical hierarchical structures. Consider the following simple example: a protein that both binds DNA and hydrolyses ATP. This protein can be equally described as a 'DNA binding protein' and as a 'catalyst' (enzyme). Consequently, this protein should be a 'child' of both 'parents' within an ontology. The presence of such multiple 'parent' situations in biology requires that they be accurately represented in a conceptual framework. DAGs, and their associated semantic relationships between the constituent nodes, meet this need.

Some of the structured controlled vocabularies being developed should be generic enough to facilitate inter-database queries for related organisms (e.g. monocots and dicots). Other ontologies would be taxon-specific but would still be able to be interrogated for inter-taxon comparisons. For example, an inter-database query for phenotypes involving the inflorescence should produce 'tassel' and 'ear' phenotypes in maize, 'panicle' phenotypes in rice and comparable inflorescence phenotypes in *Arabidopsis*. The relevant associated genomic information can then be obtained from each database for further analysis.

There is little doubt that the world-wide plant science community could benefit from having structured controlled vocabularies of terms arranged in ontologies. Furthermore, these controlled vocabularies of terms and their associated definitions will contribute towards consistent data curation and so contribute to the information management needs of the plant sciences.

### Extending the gene ontology™ paradigm

The Plant Ontology™ (PO) Consortium is extending a paradigm developed by the Gene Ontology™ Consortium [2]. The Gene Ontology™ (GO) Consortium (**http://www.geneontology.org**) has been developing ontologies and associated controlled vocabularies for several years. The objective of the GO consortium has been the development of ontologies and controlled vocabularies for three knowledge domains: the molecular function, biological process and cellular component of gene products. These ontologies are being developed for a generic

Copyright © 2002 John Wiley & Sons, Ltd. 

eukaryotic cell. The Gene Ontology™ Consortium is a paradigm for how diverse groups of researchers and database curators can share controlled vocabularies – both in terms of development (using the DAG methodology) and data sharing. Representing knowledge of biology necessitates a flexible structure – a structure that is able to change as our knowledge of biology changes. The GO paradigm readily accommodates additions and restructuring as new information is obtained. Information on new molecular functions, biological processes and cellular components can be added or updated. Several research groups have been annotating their databases according to the controlled vocabularies contained in the ontologies produced by the GO consortium. Consequently, the Plant Ontology™ Consortium is adopting and extending the GO paradigm in an effort to faithfully represent our current understanding of biology even as it changes.

### Development of plant ontologies and trait ontologies

Members of the Plant Ontology™ Consortium, and associates, are in the process of developing structured controlled vocabularies in ontologies for two large knowledge domains, namely: Plant Ontologies and Trait Ontologies. Each of these domains will comprise several sub-domains. The sub-domains, of necessity, will have components that are taxon-specific. However, while there will be taxon-specific components of ontologies, there will also be terms that will be common to all plants. For example, an ontology of anatomy for maize would include bulliform cells in the leaf, while in *Arabidopsis*, 'pavement cells' in the epidermis would be included in that taxon-specific ontology. However, both ontologies would contain the terms leaf, leaf epidermis, mesophyll etc. which are common anatomical tissues to all plants.

Consideration is being given to the use of ontogenetic and phylogenetic data and concepts in the elucidation and development of ontological relationships and the testing of the True Path Rule in the plant ontologies. The true path rule states that the pathway from a child term all the way up to its top level parent(s) must always be biologically accurate. For further information on the True Path Rule consult the General GO Guidelines in **http://www.geneontology.org/GO.usage.html**.

### Ontologies being developed

Within the Traits Ontology (**TO**, see Figure 1) will be included the following domains: agronomic traits, mutant phenotypes, phenotypes, traits, and QTL (quantitative trait loci). Genetic traits (map locations of loci of genes for each chromosome of the taxon in question) may be associated with the traits ontology.

Within the Plant Ontology (**PO**) will be included the following ontologies:

- Plant Development - This ontology will be based on published growth stage descriptors: initially for *Arabidopsis thaliana*, maize (*Zea mays*) and rice (*Oryza sativa*).
- Anatomy (incl. morphology) - This ontology deals with the cell types and tissue types in the plant body, their anatomical structure, function and location in the plant body (roots, stems, leaves, inflorescences, fruits, seeds). While the cell types and tissue types in the plant body reveal a definite structural and functional organization, their arrangement in various parts of the plant body is variable. The principal tissues of a vascular plant are grouped, on the basis of topographic continuity, into three tissue systems- the *dermal*, the *vascular* and the *fundamental* (or ground) system. The structural and functional specialization of plant anatomy, expressed in the plant's phenotype as morphology and micromorphology, is included within the *anatomy ontology*. This ontology will be initially based on *Arabidopsis thaliana* but other taxa will be included, resulting in a range of taxon-specific ontologies and subsequently ontologies that will be applicable to a generic assemblage of plants.

To explore the developing traits ontology (**TO**) consult the *Ontology Search* link at Gramene (**http://www.gramene.org/plant_ontology/**). An example of Plant Ontology (**PO**) for maize/corn leaf morphology, and specifically the ligule, is shown in Figure 2.

### Current members and associates

The Plant Ontology™ consortium is a collaboration between representatives of model organism databases and currently comprises the following

Copyright © 2002 John Wiley & Sons, Ltd.

**Figure 1.** Example of trait ontology (TO) for *sterility related trait* (TO : 0000485) from Gramene (**http://www.gramene.org**). Note that this trait illustrates the occurrence of multiple parents, a feature provided by the DAG structuring that is biologically very meaningful. Selecting any node within the ontology (online) will reveal the associated trait(s) associated with that node in the DAG structure. The [i] represents a class of semantic relationship between the concepts represented by the terms at the nodes. The [i] represents the *is a* relationship ('is an instance of'). The *is a* relationship is one of subsumption. This relationship permits refinements of concepts (represented by terms) and definitions. Other nodes in the TO at Gramene represent *part of* relationships – another class of semantic relationship between nodes and abbreviated to [p] in the online trait ontology participants: Gramene: A Resource for Comparative Grass Genomics – **http://www.gramene.org**; the International Rice Research Institute (IRRI – **http://www.irri.org**) associated with The International Crop Information System (ICIS) database (**http://www.cgiar.org/icis/**); MaizeDB (**http://www.agron.missouri.edu**) and the Maize Mapping Project (**http://www.cafnr.missouri.edu/mmp/**). The *Arabidopsis* Information Resource (TAIR - **http://www.arabidopsis.org/**) is closely associated with the collaborative efforts of the Plant Ontology™ Consortium. These collaborations are focusing on using and extending the GO paradigm to the very pressing need for ontology and controlled vocabulary development for plant-based databases. The GO paradigm is effectively described in the *General Documentation* at **http://www.geneontology.org/GO.doc.html**, and in The Gene Ontology Consortium 2000 [1], available at: **http://www.geneontology.org/GO_nature_genetics_2000.pdf**. A similar 'General Documentation' document is in the process of being developed by the Plant Ontology™ Consortium.

### Others interested

Database representatives of other plant-based databases (e.g. UK CropNet – **http://ukcrop.net**; National Institute of Agrobiological Sciences – **http://www.nias.affrc.go.jp/index_e.html**; Max Planck Institute of Molecular Plant Physiology – **http://www.mpimp-golm.mpg.de**; International Potato Center – **http://www.cipotato.org**; Virginia Bioinformatics Institute – **http://www.vbi.vt.edu**; John Innes Centre – **http://www.jic.bbsrc.ac.uk**) and plant scientists

Copyright © 2002 John Wiley & Sons, Ltd.

**Figure 2.** Example of plant ontology (PO) for the ligule of *Zea mays*, providing the term name, synonym (none for ligule), definition and references for the definition. Note that the ontology has been expanded to reveal the current ontology for some of the various other nodes associated with the leaf. The [i] and [p] represent classes of semantic relationship between the concepts represented by the terms at the nodes. The [i] represents the *is a* relationship ('is an instance of') as described in Figure 1. The [p] represents *part of* relationships ('is a part of'), another class of semantic relationship between nodes [2].

associated with various taxa (e.g. *Medicago truncatula*, soybean (*Glycine max*), common bean (*Phaseolus vulgaris*), cassava (*Manihot esculenta*) and Solanaceae (*Lycopersicon* and *Solanum*)) and companies involved in genomics, have expressed interest in becoming involved in the collaborative efforts of the Plant Ontology™ Consortium.

### Online access

A website for the Plant Ontology™ Consortium is to be developed. The URL is: **http://www.plantontology.org**. Besides the development of taxon-specific data repositories for associated controlled vocabularies and ontologies (e.g. Gramene, MaizeDB, TAIR),

Copyright © 2002 John Wiley & Sons, Ltd. 

the SPRIG site at **http://bioinformatics.org** is also to be developed as a public repository for plant ontology data. [SPRIG = Specialized Plant Resources for Informatics and Genomics]

### The Plant Ontology Consortium (Alphabetical list)

Richard Bruskiewich, International Rice Research Institute (IRRI)
**http://www.irri.org**
Edward H. Coe, MaizeDB
**http://www.agron.missouri.edu**
Maize Mapping Project
**http://www.cafnr.missouri.edu/mmp/**
Pankaj Jaiswal, Cornell University/Gramene: A Resource for Comparative Grass Genomics
**http://www.gramene.org**
Susan McCouch, Cornell University/Gramene: A Resource for Comparative Grass Genomics
**http://www.gramene.org**
Mary Polacco, MaizeDB
**http://www.agron.missouri.edu**
Maize Mapping Project
**http://www.cafnr.missouri.edu/mmp/**
Lincoln Stein, Cold Spring Harbor Laboratory
**http://www.cshl.org**
Leszek Vincent, MaizeDB
**http://www.agron.missouri.edu**
Maize Mapping Project
**http://www.cafnr.missouri.edu/mmp/**
Doreen Ware, Cold Spring Harbor Laboratory
**http://www.cshl.org**

### References

1. The Gene Ontology Consortium. 2000. Gene Ontology: tool for the unification of biology. *Nature Genet* **25**: 25-29.
2. The Gene Ontology Consortium. 2001. Creating the gene ontology resource: design and implementation. *Genome Res* **11**: 1425-1433.

Copyright © 2002 John Wiley & Sons, Ltd.
